# Genome Sequences of Two *Pseudoalteromonas* Strains Isolated from the South China Sea

**DOI:** 10.1128/genomeA.00305-14

**Published:** 2014-04-17

**Authors:** Zhenshun Zeng, Shikun Dai, Yunchang Xie, Xinpeng Tian, Jie Li, Xiaoxue Wang

**Affiliations:** Key Laboratory of Tropical Marine Bio-resources and Ecology, South China Sea Institute of Oceanology, Chinese Academy of Sciences, Guangzhou, China

## Abstract

Two *Pseudoalteromonas* strains, SCSIO 04301 and SCSIO 11900, were isolated from the South China Sea, and both strains form biofilms. Here we present the draft genome sequences of these two strains, which will aid the study of marine microbes that are adapted to marine sediments or are associated with eukaryotic hosts.

## GENOME ANNOUNCEMENT

*Pseudoalteromonas* (*Gammaproteobacteria*, *Alteromonadales*, *Alteromonadaceae*), a genus of *Gammaproteobacteria*, was differentiated from the *Alteromonas* genus in 1995 according to the difference in small-subunit rRNA gene sequences (1). *Pseudoalteromonas* is widespread in marine environments and has become an organism of interest to the fields of ecological and pharmaceutical sciences due to its influence on biofilms and its ability to synthesize bioactive molecules (2). So far, draft genome sequences of over 40 *Pseudoalteromonas* strains have been released to public databases, and three strains have complete whole-genome sequences.

Here we present the genome sequences of two *Pseudoalteromonas* strains, *Pseudoalteromonas lipolytica* SCSIO 04301 and *Pseudoalteromonas* sp. strain SCSIO 11900. SCSIO 04301 was isolated from sediment at 63 m deep in the South China Sea (18°0′N, 109°42′E), and SCSIO 11900 was isolated from the surface mucus layer of the coral at 4 m deep in the South China Sea (18°13′N, 109°28′E). The 16S rRNA sequences of SCSIO 04301 share 100% similarity with *Pseudoalteromonas lipolytica* LMEB 39 (3), while SCSIO 11900 is most closely related to the deep-sea sediment-adapted strain *Pseudoalteromonas* sp. SM9913 (4). The SCSIO 04301 and SCSIO 11900 strains are nonpigmented. Similar to other *Pseudoalteromonas* strains, both strains produce large amounts of extracellular polymeric substances and form pellicles and biofilms.

Sequences were obtained using the Illumina HiSeq 2000 sequencing platform; 680 Mb and 677 Mb of clean data were produced for the SCSIO 04301 and SCSIO 11900 genomes, respectively. SOAPdenovo (version 1.05) (5, 6) was used to assemble the reads after filtering. The assembled result was then locally assembled and optimized according to paired-end and overlap relationships. The sizes of the genomes were estimated by k-mer analysis, the GC contents were calculated by GC-depth analysis, and the protein-coding open reading frames (ORFs) were predicted by Glimmer (7, 8) (version 3.02). For RNA prediction, rRNA was predicted by the rRNA database and RNAmmer (9) (version 1.2) and tRNA was predicted by tRNAscan (10).

Based on the assembled results, the genome size of SCSIO 04301 is 4.7 Mb and the GC content is 41.26%. The number of scaffolds is 12 and the total coverage over the genome is 145-fold. For SCSIO 11900, the genome size is 3.7 Mb, with a GC content of 40.45%. The number of contigs is 22, and the total coverage over the genome is 183-fold. A total of 4,215 ORFs, 95 tRNAs, and 6 rRNAs are predicted for SCSIO 04301, and a total of 3,515 ORFs, 89 tRNAs, and 26 rRNAs are predicted for SCSIO 11900. Two chromosomes were found for both SCSIO 04301 and SCSIO 11900, and the circularizations of the small chromosomes were confirmed again by PCR and subsequent DNA sequencing. A comprehensive study combined with comparative genome analysis and phenotypic analysis is under way to explore the relationships between genetic variation and phenotypic variation of *Pseudoalteromonas* strains in different ecological niches.

### Nucleotide sequence accession numbers.

This whole-genome shotgun project has been deposited at DDBJ/EMBL/GenBank under the accession numbers JDVB00000000 for SCSIO 04301 and JEMJ00000000 for SCSIO 11900.

## References

[1] Gauthier G Gauthier M Christen R 1995 Phylogenetic analysis of the genera *Alteromonas*, *Shewanella*, and *Moritella* using genes coding for small-subunit rRNA sequences and division of the genus *Alteromonas* into two genera, *Alteromonas* (emended) and *Pseudoalteromonas* gen. nov., and proposal of twelve new species combinations. Int. J. Syst. Bacteriol. 45:755–761. 10.1099/00207713-45-4-755 7547295

[2] Holmström C Kjelleberg S 1999 Marine *Pseudoalteromonas* species are associated with higher organisms and produce biologically active extracellular agents. FEMS Microbiol. Ecol. 30:285–293. 10.1111/j.1574-6941.1999.tb00656.x 10568837

[3] Xu XW Wu YH Wang CS Gao XH Wang XG Wu M 2010 *Pseudoalteromonas lipolytica* sp. nov., isolated from the Yangtze River estuary. Int. J. Syst. Bacteriol. 60:2179–2181. 10.1099/ijs.0.017673-0 19897616

[4] Yan BQ Chen XL Hou XY He H Zhou BC Zhang YZ 2009 Molecular analysis of the gene encoding a cold-adapted halophilic subtilase from deep-sea psychrotolerant bacterium *Pseudoalteromonas* sp. SM9913: cloning, expression, characterization and function analysis of the C-terminal PPC domains. Extremophiles 13:725–733. 10.1007/s00792-009-0263-1 19544039

[5] Li RQ Li YR Kristiansen K Wang J 2008 SOAP: short oligonucleotide alignment program. Bioinformatics 24:713–714. 10.1093/bioinformatics/btn025 18227114

[6] Li R Zhu H Ruan J Qian W Fang X Shi Z Li Y Li S Shan G Kristiansen K Li S Yang H Wang J Wang J 2010 De novo assembly of human genomes with massively parallel short read sequencing. Genome Res. 20:265–272. 10.1101/gr.097261.109 20019144 PMC2813482

[7] Delcher AL Harmon D Kasif S White O Salzberg SL 1999 Improved microbial gene identification with GLIMMER. Nucleic Acids Res. 27:4636–4641. 10.1093/nar/27.23.4636 10556321 PMC148753

[8] Delcher AL Bratke KA Powers EC Salzberg SL 2007 Identifying bacterial genes and endosymbiont DNA with Glimmer. Bioinformatics 23:673–679. 10.1093/bioinformatics/btm009 17237039 PMC2387122

[9] Lagesen K Hallin P Rødland EA Staerfeldt HH Rognes T Ussery DW 2007 RNAmmer: consistent and rapid annotation of ribosomal RNA genes. Nucleic Acids Res. 35:3100–3108. 10.1093/nar/gkm160 17452365 PMC1888812

[10] Lowe TM Eddy SR 1997 tRNAscan-SE: a program for improved detection of transfer RNA genes in genomic sequence. Nucleic Acids Res. 25:955–964. 10.1093/nar/25.5.0955 9023104 PMC146525

